# The Use of Compressed Air in Operative Dentistry

**Published:** 1900-03

**Authors:** S. Freeman

**Affiliations:** New York


### THE USE OF COMPRESSED AIR IN OPERATIVE DENTISTRY.[1]

BY DR. S. FREEMAN, NEW YORK.

At the request of the chairman of the Executive Committee' Dr. Charles O. Kimball, I have the pleasure of presenting to your Society a paper upon "The Use of Compressed Air in Operative Dentistry," and without any further preliminaries I will describe the apparatus that is used and its mode of application.

Permit me to explain the methods of producing compressed air and the manner of conducting the same to your operating-chair.

It is obtained by means of a suction-pump which sucks in the air and forces it through a pipe to a reservoir, as it is necessary for our purpose to have a continuous and considerable current. I would not advise the use of a hand-pump or small cylinder, as in employing them you will find that both the air and the operator become quickly exhausted. I would therefore recommend that which is known as the champion beer-pump, or the compound pump of the same manufacture, for I find both the simplest in construction, as well as the most satisfactory air compressors on the market to-day.

Before placing the pump in your office, ascertain how many pounds of water-pressure you have; if, as in my office, you have only twenty-five pounds, the champion pump will not furnish, contrary to the claim of the manufacturers, the same amount of air-pressure as water, and there is, as I have discovered, a loss of a few pounds; such being the case, the compound pump (although a few pounds of pressure are also lost) is preferable, as it is frequently necessary to have the air at a pressure of forty to fifty pounds to the square inch.

This pump is connected with the reservoir, which is a tank containing eighteen gallons of air tested to cne hundred and fifty pounds pressure to the square inch; from this runs a quarter of an inch block-tin pipe, with branches to my laboratory and operating room; to this pipe I have attached a regulator and a gauge, the regulator being nothing more than a screw-valve; as you loosen the screw you close the valve, and *vice versa.*

From the regulator a pipe leads to the gauge, which indicates the number of pounds pressure.

---

[1] Read before the New York Institute of Stomatology, November 9, 1899.

I use the eighty-pound gauge, as it enables me to ascertain the maximum amount of pressure to my office.

The pressure generated by the compound pump is sometimes so great that a lower gauge will not register it.

To the gauge is connected a distributing pipe with three small cocks to attach the rubber tubes and the cut-off. The tubes should be of heavy rubber, so as to withstand a high air-pressure. The cut-offs close automatically, and you may use the somewhat antiquated expression, touch the button and the air will do the rest.

In the practice of dentistry it is absolutely necessary to have the mouth in an aseptic condition; to produce this effect, no doubt many of you use the hand-atomizer, which, by the continuous pressure of the bulb, causes a slight cramp in the hand; with the compressed-air apparatus we do away with this work; you have the tube steadied and can thoroughly cleanse the mouth or any cavity. I derive excellent results from the following prescription:

℞ Borine, 1 part;
Dioxide med., 3 per cent., 2 parts;
Water, 1 part.

This makes an agreeable mouth wash applied with a spray.

No doubt you have frequently met cases where you would prefer to place the rubber dam on, but, owing to the patient's inability to breathe through the nostrils, have been compelled to send your patient away, or otherwise work at a great disadvantage.

How often have you cast about for a remedy? It is a simple one,—a two per cent. solution of cocaine placed in an atomizer attached to your compressed-air apparatus, gauged at ten to fifteen pounds pressure; and the spray then thrown into the nostrils will invariably relieve that posterior nasal catarrh or reduce the swollen tissues of the nares to such an extent that you can proceed with your work in a few minutes, without any inconvenience to your patient. In using the spray for this purpose, have your patient sitting upright in the chair, the head inclining slightly forward. Insert the tube horizontally into the nares, and do not apply over ten to fifteen pounds of air-pressure, as otherwise you may set up an irritation of the middle ear.

Where we have nausea arising from taking impressions, placing on the rubber dam, or even pregnancy, a two per cent. solution of cocaine blown directly up the nostrils, so as to have the fluid in contact with the olfactory nerves, will often relieve the severest case of retching. In using the spray in the nostrils you must not permit your patient to blow their nose for several minutes, as otherwise the fluid may be blown into the middle ear, and subsequent irritation arise.

In stomatitis of the various kinds, the spray employed with a high pressure produces excellent results.

In aphthous stomatitis, which usually requires about a week to ten days to heal, I have succeeded in obtaining a cure in two days.

Probably the citation of the following case will enable you to better understand the value of this agent.

Mr. H., aged seventeen years, applied to me for treatment, May 7, 1895, suffering from large grayish patches situated on the lower and upper lips. These patches were very painful; I applied the following prescription,—

℞ Pyrozone med., 3 per cent.;
Borine, āā.

with my atomizer, under forty pounds pressure; in about two minutes it produced bleeding of the sores; washing away the grayish patch, and you could see the ebullition of the pyrozone, leaving escharotic spots similar to those produced by the application of the caustic pyrozone. I gave the patient the mouth wash recommended by Dr. Sudduth, of pyrozone and soda mint; the patient reported on the ninth day of May having no signs of the patches. This method of treatment was not painful and proved very efficient, as I have used it since in similar cases with uniformly good results.

Before applying any medicines to the gums, it is always necessary to have a dry surface, so that the medicament may be readily absorbed and not distribute itself over the surface of the tissue.

I found it somewhat difficult to obtain a dry condition of the gums in the posterior portion of the mouth before applying this apparatus. Now I find it a very simple matter. By drawing back the corners of the mouth with a napkin or piece of cottonoid, and throwing the compressed air directly to the spot, it only requires a few seconds to procure the desired condition of the mucosa, and upon applying your medicines you get immediate absorption.

Now, I again have recource to the air-pressure, which seems to drive the medicines deep into the tissues.

In periostitis I prefer to use the cold air current, which in itself gives relief to the patient.

In this manner the application of counter-irritants, sedatives, and local anæsthetics is made easy. With cocaine you do not obtain as deep an anæsthesia as you do by the hypodermic injection of the drug; but with a four per cent. solution I was enabled to lance abscesses within twenty to thirty seconds without pain.

In the treatment of the antrum, no doubt the report of an interesting case of this disease will not be amiss, and will thoroughly explain my method.

Miss M., aged fourteen years, applied to me for treatment on May 7, 1896. Upon examination I found an irritation of the gum between the second superior left bicuspid and first molar, caused by a piece of wooden tooth-pick, which she was unable to dislodge; after removal of the irritant, I painted the gum with a mixture of tincture of iodine, aconite capsicum, and chloroform. On June 26 the patient complained of a discharge of pus and swelling of the left side of the face; I discovered a fistula situated about a sixth of an inch above the border of the alveolus, between the bicuspid and molar.

The second bicuspid was a perfectly sound tooth, the first molar had a small amalgam filling, and upon opening the molar the pulp was seen to be in a healthy condition. I extracted the molar, which was followed by a flow of pus, and after spraying the parts with pyrozone med., three per cent., at a pressure of forty to fifty pounds, found an opening into the antrum about a sixteenth of an inch in diameter; upon removing all the necrosed tissue and cleaning the parts with the spray, I took an impression and had Dr. F. J. McLaran make a small rubber plate to thoroughly close up the orifice. This case was treated daily for a week with pyrozone med., three per cent., and borine, spraying the medicament at a pressure of fifty to sixty pounds, using a Davidson atomizer and sprinkling the extension on the plate with powdered aristol.

I permitted the patient to leave the city, instructing her how to keep the parts clean and keep the plate in an aseptic condition. On July 28 she called, when it was necessary to remove one-half of the extension of the plate. On August 15, the cavity was entirely closed with healthy tissue. September 1, I discharged my patient thoroughly cured.

In the treatment of pyorrhœa alveolaris it is absolutely essential that every particle of deposit, of whatever form, shall be removed, and that the *débris* shall be thoroughly washed away, leaving no particles to be ulcerated; to do this it requires a vigorous stream of water.

Attached to my compressed-air apparatus, at fifty to sixty pounds pressure, I use the Davidson spray tubes (filled with pyrozone med., three per cent., and borine), which throw out such copious spray that it lifts the gum-tissues away from the teeth, introducing the medicament directly to the seat of trouble, forcing away all foreign particles and giving a clean aseptic condition of the parts.

I employ these spray tubes, attached to my compressed-air apparatus, for introducing medicine and washing out cavities preparatory to implanting teeth, and I may be concise and say for all wounds and diseases of the oral cavity.

Having considered the advantages derived from the aid of compressed air upon the soft tissues, I will briefly state the results procured through the medium of this agent on the hard tissues, the teeth.

It is not necessary for me to cite in detail the minute anatomy of the teeth, although a thorough knowledge of the structure of the dentine is requisite to fully appreciate the ideas which I wish to convey.

I will therefore ask your attention to a short review of the minute structure of this tissue. A great portion of the tooth consists of dentine, which is composed of an organic matrix richly impregnated with calcareous salts.

This matrix is everywhere permeated with parallel tubes, which run, with some deviation, in a direction at right angles to the surface of the tooth.

These tubes start by an open circular mouth upon the surface of the pulp ; thence running outward, towards the periphery of the dentine, they become smaller and break up into branches at a little distance beneath the surface of the dentine. Near the pulp they are so closely packed that there is little room between them for the matrix, while near the outside of the tooth they are more widely separated. Their diameter is also greater near the pulp cavity.

These tubes are subject to slight varicosities, and their course is somewhat interrupted by a small interglobular space.

Each tube is occupied by a soft fibril, which is continuous with an odontoblast cell upon the surface of the pulp ; of their real nature some doubts have been entertained, but I will not occupy your time by citing from different authorities.

These fibres cannot be considered nerves in the ordinary sense of the word. There cannot be any doubt but that they are media for the transmission of sensory impressions from the dentine to the pulp, and that the peripheral sensitiveness can be allayed by local applications.

Drs. Harlan, Kirk, and Truman demonstrated, by experiment, that there is an absorption of liquids by osmotic action. The former states that "thymol will diffuse in moist dentine in from three to six hours at 98.4° F.; that non-coagulants, soluble in water, diffuse readily through tooth structure, as has been shown repeatedly in experiments outside of the mouth; that oleaginous non-coagulants pass through the structure of a tooth quite slowly in the presence of water in serum albumin, and that the vaporizable portion of an essential oil will give to a substance which it permeates the characteristic odor in from three to six hours."

Professors Truman and Kirk show by experiments that coagulants will penetrate the tooth structure, and Professor Truman states "that in proportion to the coagulating power of the agent will be the penetrating force independent of gravitation."

These statements have awakened the profession, and provoke no little discussion, although the controversial period is usually only a passing, and never the most fruitful, period of any truth.

After a science has gained a recognized footing, it has before it its real work to do. The question arises, What can you demonstrate?

These gentlemen have established the fact that medicines will be absorbed through the tubuli *after a certain length of time.*

Now, you will remember that the gluey yielding portion of a tooth contains water to about ten per cent. of the weight of the entire tooth; bearing this in mind, let me ask you, what can we accomplish after dehydration of the tissues, leaving entirely out of consideration the capillary attraction of these infinitesimal dentinal tubes.

It has been demonstrated that when the water is removed from a tooth, the normal function of transmitting impressions seems to be modified. This desiccation can be accomplished by several agents,—heat, cold, and chemicals.

We know that heat or cold will produce pain, and in the application of either we should proceed with extreme caution.

I use a hot-air syringe similar to the S. S. White's No. 30, only this has twice as large a cylinder. The chamber is filled with carbon, which is found to be one of the best materials for retaining caloric, and only requires a few minutes over a Bunsen burner flame to accomplish the requisite amount of heat; with this syringe attached to the compressed-air apparatus, you can so regulate the flow of air that in from one-half to a minute you have your tooth thoroughly dry; then introduce your medicine, heated to about 95° F., again applying your warm-air current with about forty pounds pressure, and you will be able to excavate your tooth without pain, nor will you have any subsequent irritation. With this method it is not necessary to employ acids in introducing your cocaine, nor is much of your valuable time wasted waiting for the absorption of the medicament.

You can also use the cataphoric action with cocaine, but I would first advise the dehydration of the dentine before applying the current, for in that way you gain better results in a shorter time.

In the bleaching of teeth, I find that by the application of hot air at a high pressure, I am able to produce the required condition in one-half the usual time, as you rapidly evaporate the pyrozone twenty-five per cent., and force it into the tubuli.

It is necessary to be cautious in bleaching teeth so as not to get them too white, as you will frequently observe them somewhat lighter colored on the following day.

In making a tooth perfectly aseptic, the same method of applying your drug as I have recommended in inducing anæsthesia, will give you a reliable aseptic condition of both tooth and root.

For drying out root-canals, use this small point on your syringe, and you can carry heated air directly in the canal, and with a high pressure can force your antiseptic through the canaliculi and tubules, rendering the tissues thoroughly aseptic.

In setting in pivot teeth, we often find it difficult to carry the cement to the upper portion of root. In forcing it into the root-canal with a dry instrument, upon withdrawal of the latter the cement is found adherent to it, and not to the walls of the root; but with a high pressure you can force it into every irregularity of the root, while at the same time the compressed air will dry all the surrounding tissue, and you avoid the necessity of wiping away the moisture excreted by the mucous glands.

Where heat is caused by drilling, using sand-paper disks or strips, by applying the cold-air current you can work without any interruption, for it keeps the tooth at about the normal temperature, causing no irritation. Dr. Oscar Adelberg recommends the use of nitrous oxide gas for this purpose, but compressed air you will find preferable and inexpensive.

Gentlemen, I will not occupy any more of your valuable time in enumerating the many advantages these appliances have over the ordinary hand-pressure instruments, but will ask you to try them; and I am certainly satisfied that you will never regret having placed this apparatus in your office.

